# #lowbackpain on TikTok: A New Frontier for Orthopaedic Medical Education

**DOI:** 10.5435/JAAOSGlobal-D-23-00181

**Published:** 2024-04-19

**Authors:** Sazid Hasan, Conner D. Ahlgren, Matthew Lipphardt, Alexandria Chrumka, Razeen Zaman, Ridwana Khan, Muhammad Waheed, Devan O. Higginbotham, Ehab Saleh, Scott A. McCarty

**Affiliations:** From the William Beaumont Hospital, Royal Oak, MI (Mr. Hasan); Oakland University William Beaumont School of Medicine, Rochester, MI (Mr. Hasan, Dr. Saleh); the Department of Orthopaedic Surgery, Beaumont Hospital, Royal Oak, MI (Dr. Ahlgren, Dr. Lipphardt, Dr. Chrumka, and Mr. Zaman); the University of Michigan College of Literature, Science and the Arts, Ann Arbor, MI (Ms. Khan); and the Department of Orthopaedic Surgery, Detroit Medical Center, Detroit, MI (Dr. Waheed, Dr. Higginbotham, and Dr. McCarty).

## Abstract

**Introduction:**

Low back pain has become a substantial health problem in all developed countries. Many healthcare professionals and content creators have begun sharing their treatment methods and opinions through social media, especially the video-based platform TikTok. TikTok has been downloaded more than 2.6 billion times with over a billion daily users. Its influence on public health makes it imperative that information be accurate and safe. This study aims to analyze TikTok's most popular content on lower back pain and how orthopaedic surgeons contribute on this growing platform.

**Objectives:**

To analyze TikTok's most popular content on lower back pain and how orthopaedic surgeons are and can contribute on this growing platform.

**Methods:**

A TikTok search conducted on April 22, 2023, using the terms ‘#lowerbackpain'and ‘#lowbackpainrelief,’ resulted in numerous videos, 100 of which met inclusion criteria. Videos were included if they were related to the content, had more than 1000 views, were in English, and were not duplicates. Video characteristics were recorded and evaluated for quality by two reviewers using DISCERN. A two-sample *t*-test was used to assess differences.

**Results:**

Overall, the top videos on lower back pain had an average of 2,061,396 views, with a mean DISCERN score of 34. The mean total DISCERN score was 36 and 34 for physicians and nonphysicians, respectively, while the video by the orthopaedic surgeon (n = 1) scored 31. The most recommended treatments included at-home exercises (n = 75) and visiting a chiropractor (n = 4).

**Conclusion:**

We find that the information presented by nonphysicians offered quick, at-home fixes to medical problems without offering any research or proven data to support their claims. We cannot overlook Tiktok's immense influence in the realm of orthopaedic health as it has become a sphere of information dissemination and education. Thus, we suggest that there is not necessarily a need for a greater number of surgeons and/or resident physicians to involve themselves on the platform, but rather the involvement of governing bodies and spine societies to put out position statements for our patients.

Low back pain (LBP) is a prevalent, dilapidating condition that results from a diverse array of etiologies. Approximately 50 to 80% of adults experience a component of LBP at some point in their lives, with some estimates reporting this number to be as high as 84%.^1^ Its prevalence is compounded by the resulting morbidity, with LBP reported to be the leading cause of years lived with disability globally, contributing to 10.7% of years lived with disabilities recorded between 1990 and 2010.^2^ In the ever-evolving landscape of health information dissemination, social media platforms have become instrumental in shaping public perceptions and understanding. Among these, TikTok stands out as a powerful influencer in the digital health realm.

Social media has become the pervasive medium for disseminating information, with 72%^3^ of the population participating in some capacity. With its increasing popularity, social media has not surprisingly permeated into the medical field. 75% of patients look to the Internet for information on providers and disease processes before presenting to care institutions.^4^ This is especially true in the field of orthopaedics, as orthopaedic patients often use the Internet and social media ^5^ exclusively for educational material. The rising influence of social media can be a powerful tool for clinicians, as appropriate social media mobilization has had positive impacts on patient outcomes.^6^ TikTok is currently the fastest-growing social media platform with more than three billion downloads to date.^7,8^ The shorter video spans on this platform combined with the user's tailored video recommendations makes it the ideal platform for maximizing the dissemination and retention of appropriate information.^6^ However, given its open platform, medical information presented on TikTok can come from nonvetted sources, enabling the potential propagation of unvalidated information.^9,10^ An analysis of neurosurgical content on TikTok demonstrated that content on the platform is vastly subject to biases and may relay inaccurate medical information.^7^ Furthermore, provider involvement is scarce on the app, with one study demonstrating that 0 of 1,231 practicing members of the Pediatric Orthopaedic Society of North America (POSNA) had accounts on the popular platform.^11^ The limited involvement of qualified providers, combined with the unvetted nature of content, highlights the potential risks of this platform in the context of possible medical disinformation. This study recognizes TikTok as not merely a social networking platform but a dynamic space where health education intersects with entertainment, offering a unique avenue for engaging a broad audience.

Analysis of social media content regarding back pain has been conducted previously in regard to YouTube and other platforms. Our study is the first to assess the quality of lower back pain and spine surgery content on the popular platform, TikTok. TikTok serves as a platform of interest to both patients and physicians because of its broad scope of content, reach, and ease of use. However, we seek to highlight the potential shortcomings of this content on this particular platform with the hopes of encouraging much-needed provider involvement in this ever-growing medium. Furthermore, by delving into TikTok's role in disseminating information, the research aims to inform healthcare professionals, governing bodies, and the general public about the platform's effect on orthopaedic health education.

## Methods

A comprehensive, cross-sectional analysis of LBP content on TikTok was carried out on April 22, 2023, using the hashtags ‘#lowerbackpainrelief’ and '#lowerbackpain'. The search resulted in 173 videos, and after applying our inclusion criteria, based on preexisting models,^8,9^ 100 videos were selected for our study. These criteria specified that videos must contain relevant content to LBP, have achieved more than 1,000 views, and be delivered in the English language. Duplicate videos or those that failed to meet the criteria were excluded, leading to the removal of 73 videos.

Each selected video was subjected to detailed descriptive statistics. Metrics such as video likes, comments, view count, uploader type, uploader sex, and physician specialty, if applicable, were carefully recorded. The quality of the content was assessed by two of the authors (RZ & RK) using the DISCERN tool, a well-validated instrument designed for the appraisal of consumer health information. This tool ranks the content quality on a scale of one (poor) to five (excellent) across 15 questions, allowing for a nuanced evaluation of the videos.

In addition to these measures, to mitigate potential bias from the TikTok algorithm, a new account was created specifically for this study. This ensured that the selected videos did not reflect a single author's preferences and that the content was representative of what a new user might encounter on the platform. All videos were queued, watched, and assessed in a single sitting to further reduce bias and maintain consistency in the assessment process.

Inter-rater reliability was calculated using Pearson correlation, ensuring consistency in the ratings assigned by the two independent evaluators. Numerical data were summarized as mean ± standard deviation while categorical variables were expressed as n (%). An unpaired, independent two-sample *t*-test was used to analyze the differences in mean DISCERN scores between physician and nonphysician groups. Statistical significance was set at *P* < 0.05, and all analyses were conducted using Microsoft Excel v. 2103 (Microsoft Corporation, Redmond, Washington).

## Results

A total of 100 videos meeting inclusion criteria were included in our final analysis. When assessing sex distribution, most content creators were male (n = 72, 72%) while a smaller number were female (n = 28, 28%). Content creators were most commonly nonphysician medical providers (n = 81, 81%), followed by physicians (n = 12, 12%) and private companies (n = 7, 7%). The views for physicians had a mean value of 3,090,425 ± 9,048,992 while nonphysicians had a mean value of 2,281,300 ± 6,010,344 views (Table 1). Surprisingly, private companies averaged a total of 1,130,115 ± 2,031,982 views. Despite the larger sample size for nonphysicians, physicians had a higher mean DISCERN score with a value of 36 ± 5 while nonphysicians had a value of 34 ± 5 (*P* > 0.05).

**Table 1:** Study Demographics and Video Statistics

TikTok	Race	Total No. of Videos	Average No. of Views	Average No. of Likes	Average No. of Comments	Recommended Treatment/Medication
Physician average	White: 6	12	3,090,425	119,552	478	Stretches: 6, surgery: 1, kinesiology: 1, posture: 1, exercises: 1, seek care by a physician: 2
	Black: 0					
	Asian: 6					
	Hispanic: 0					
	Other: 0					
SD			9,048,992	372,198	9,861	
Nonphysician average	White: 66	88	2,281,300	165,554	1,278	Stretches: 38, exercise: 28, acupuncture: 2, chiropractor: 4, yoga: 2, pressure points: 2, posture: 2, chakras: 1, words of affirmation: 1, regenerative medicine: 1, seeking medical help: 1,
	Black: 4					
	Asian: 16					
	Hispanic: 0					
	Other: 2					
SD			6,010,344	524,138	4,055	

SD = standard deviation

**Figure 1:**
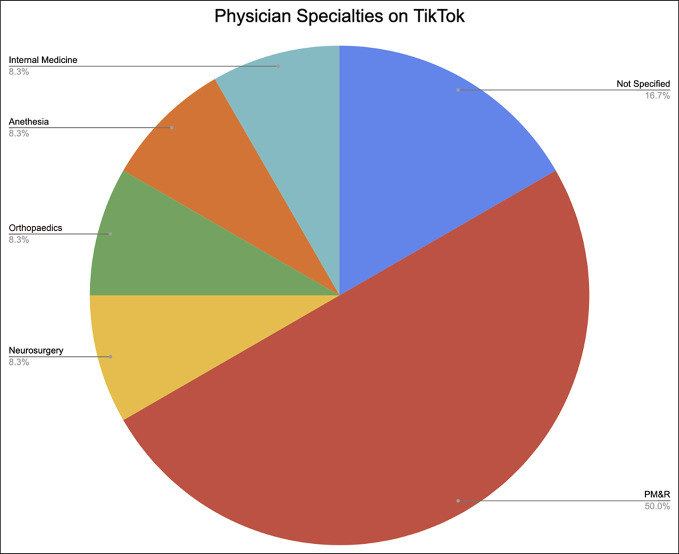
Pie chart showing physician content creator distribution.

Most physician content creator videos (n = 12) were created by PM&R physicians (n = 6, 50%) while orthopaedic and neurosurgeons each accounted for just one video (8.3%; Figure 1). In addition, 84% of the TikTok videos included suggested treatments for LBP which consisted of at-home exercises (n = 75, 75%), visiting a chiropractor (n = 4, 4%), and seeking professional medical help (n = 5, 5%). However, owing to the nature of TikTok having short videos to keep engagement, more than 95% of the videos observed did not mention any risks associated with each treatment nor did the uploaders provide evidence to support their treatment choices making it difficult for TikTok users to assess the accuracy and safety of the suggested treatments.

Of the video types included in the experiment, most them were described as home remedies (n = 78) such as various exercises and stretches or educational content (n = 8) which included daily habits to reduce and prevent LBP. This is followed by treatment advertising (n = 6) such as seeking medical or chiropractic assistance and personal experience (n = 5; Table 1). The DISCERN scores for home remedies were significantly higher than those for treatment advertising, despite averaging the lowest number of views. In addition, the home remedies averaged the highest number of likes (158,935 ± 22,250).

**Figure 2:**
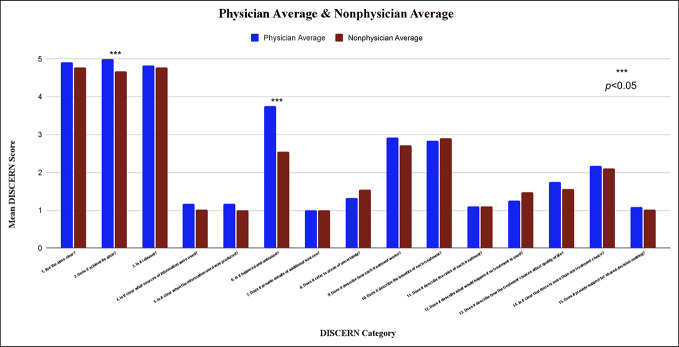
Graph showing comparison of DISCERN scores between physicians and nonphysicians.

Creators on Tiktok scored highest (scores of 4 to 5) on DISCERN questions 12 (“Does it achieve its aims?”) and 3 (“Is it relevant?”; Figure 2). Whereas scores on questions 4 (“Is it clear what sources of information were used?”), 5 (“Is it clear when the information used was produced?”), 6 (“Is it balanced and unbiased?”), 7 (“Does it provide details of additional sources?”), 8 (“Does it refer to areas of uncertainty?”), and 11 (“Does it describe the risks of each treatment?”) were lower (scores 1-2). Physicians scored higher on the lowest-scoring questions with a few private companies scoring higher as well. Overall DISCERN scores were not significantly different between physicians and nonphysicians (*P* > 0.05).

## Discussion

TikTok has become a new frontier in the realm of human interaction and as such represents a new medium through which consumers and potential patients engage with medical information. Videos tailored on the basis of an individual rather than audience have given way to the phenomenon of TikTok addiction, behavior marked by deep immersion and increased interaction with the platform.^12^ This has partially been equated to the platform's dynamic algorithm and the short video application which allows information to be conveyed in a concise and specific way.^12,13^ This makes TikTok an ideal environment for medical education because it aligns well with the emerging trend of "microlearning," which is rapidly replacing standard long-format lectures.^14^

The literature demonstrates that physicians across multiple medical disciplines have taken to the platform to educate the public on various topics encoded by their topic's respective hashtags.^3,7,8,10,15^ Based on our screened studies and researched hashtag, we found a significant lack of orthopaedic surgeon content creators. PM&R physicians accounted for 50% of all physician videos (6 of 12 physician videos); however, we found only one orthopaedic surgeon content creator. This may not be as surprising as many think given that 56.2% of full-time orthopaedic surgeons are between age 40 and 59 years with the average age of a surgeon to be 56.5 years.^16,17^ This is a sharp contrast to the average TikTok creator falling between 18 and 24 years.^18^ In addition, Klein et al^16^ showed that surgeons worked about 70.3 hours per week with 75% of surgeons reporting that they did not have as much time for their personal lives as they would like. While the authors recognize the busy, productive lives of orthopaedic surgeons, we cannot overlook TikTok's immense influence in the realm of orthopaedic health because it has become a sphere of information dissemination,^3^ advertisement of treatment, and a platform to educate potential patients. However, although many may suggest that encouraging more physicians to participate on the platform may be a solution, we find that this may not be the case. Entertainment skews the realm of professionalism, and therefore, we encourage our current medical governing bodies and spine societies to put out position statements for our patients. It may be within reason that they themselves get involved on the platform because it will allow for the dissemination of standardized, accepted guidelines and practices.

Given the nature of TikTok and the lack of requirements for content citing, peer-review, and overall screening of information, the potential for harmful misinformation or poor-quality of information being disseminated is extremely high. Misinformation lacking scientific evidence has been reported for various medical conditions on the platform. Patients, family members, or caregivers without a medical background can have difficulty verifying and ensuring the credibility of information posted on social media. O'Sullivan et al^19^ showed that of their 27 videos that met their criteria, only 22.2% of videos contained information that could also be found in European Association of Urology guidelines. In addition, none of their videos contained any cited evidence to legitimize statements that were being made by content creators. They also noted that of the 61 videos the study sampled, healthcare practitioners contributed the most content at 47.54% (n = 29) of the videos. However, they noted that many of the videos were misinformative with many content creators using outdated data.^19^ In our study sample, physicians typically advised patients of stretching, surgery, education, and exercises, treatment modalities that are shown to improve symptoms of lower back pain in the literature.^20,21^ However, in the nonphysician group, videos often discussed topics including balancing of chakras, words of affirmation, acupuncture, and other treatment modalities that are not supported by the literature. Given the fact that these nonphysician videos encompassed most of our sampled videos (n = 81, 81%), we can conclude that the rate of unsupported information dissemination is much higher than the information presented by physician creators. This discrepancy may lead to the dissemination of misinformation to a large target audience and sway their opinion of medical information and potential treatment modalities. We affirm the need for the practice of evidence-based medicine and recommend that physicians continue to do so in their videos to increase engagement with appropriate evidence-based recommendations.

Poor-quality information may prove to be disadvantageous to both physician and nonphysician content creators alike as information quality has been shown to positively influence user enjoyment and concentration during their time spent on the platform.^12^ When objectively grading the content quality of physician vs nonphysician videos, we find that the total DISCERN score was 36 ±5 and 34 ± 5 for physicians and nonphysicians, respectively (*P* > 0.05), and the video by the orthopedic surgeon scored a 31. A DISCERN score between 27 and 38 is considered “poor”,^22^ emphasizing the need for an increase in the quality of their content. When assessing our data, we found that statistically significant differences between physicians and nonphysicians were particularly in the categories of “does it (video) achieve its aims” and “is it balanced or unbiased”. The variance in health-related content shared by doctors and nonprofessionals on platforms such as TikTok arises from diverse factors. Doctors, armed with medical expertise, tend to prioritize evidence-based information, often using specialized language that might be less accessible. Their motivation centers around educating the audience and dispelling health myths, but they may grapple with balancing accuracy and audience engagement. By contrast, nonprofessionals adopt a more relatable communication style, often drawing from personal experiences and wellness trends. Their content creation is influenced by entertainment value and trends, sometimes at the expense of scientific accuracy. The platform's dynamics, algorithm rewards, and the perceived responsibility associated with disseminating health information contribute to the dichotomy. Doctors, cognizant of potential risks, approach content creation cautiously, whereas nonprofessionals, perhaps unaware of the broader implications, may prioritize attention and engagement.

As flag bearers of evidence-based medicine, citing information is imperative when conveying information. However, videos by providers and nonproviders alike lacked appropriate citation as is apparent with the low DISCERN score assigned to this category. While citing information sources would be beneficial on the objective score criteria, it may be unlikely that viewers actually access the information as open access to journals/articles is estimated to be as low as 20%.^23^ In addition, this may be challenging for physicians even through the omnipresent “microlearning” phenomenon as many topics require long, complex explanations beyond the short-term quick information nature of the platform. While TikTok has recently updated its app to allow people to post videos up to three minutes long, most of those on TikTok tend to view videos from 10 to 60 seconds in length.^24^ In addition, it is important to recognize that the reading level of those living in the United States stands at around eighth-grade level,^5^ far lower than the level of competency required to appreciate peer-reviewed content. Physician content creators will benefit greatly from tailoring their videos to be short, visually appealing, and factual while also making sure to be comprehensible to the average viewer.

## Limitations

Our study has multiple limitations, the first is in regard to the cross-sectional nature of our study which eliminates our ability to establish any causal relationships. Second, the DISCERN scoring system does not touch on the accuracy or validity of the information being presented. In addition, we are only studying one platform, thus we are unable to generate a general commentary on the state of LBP information throughout social media. We are also thus unable to analyze the way video content changes because of TikTok-specific attributes such as its popularity or short video length. Furthermore, our study was limited to videos in English; therefore, our findings cannot be extended to other popular languages in TikTok videos such as Spanish or Chinese. Furthermore, our limited sample size of 100 videos on a platform tailored toward billions of users may not be fully representative of the available content on LBP available to users. Finally, multiple other methods of consumer health information appraisal which are available (in addition to DISCERN) were not used.
